# Efficient algorithms for reconciling gene trees and species networks via duplication and loss events

**DOI:** 10.1186/1471-2164-16-S10-S6

**Published:** 2015-10-02

**Authors:** Thu-Hien To, Celine Scornavacca

**Affiliations:** 1 ISEM - Université de Montpellier, CNRS, IRD, EPHE, Place Eugène Bataillon, 34392, Montpellier, France; 2 Institut de Biologie Computationnelle (IBC), 95 rue de la Galera, 34095, Montpellier, France

**Keywords:** tree reconciliation, gene evolution, phylogenetic, parsimony, phylogenetic networks

## Abstract

Reconciliation methods explain topology differences between a species tree and a gene tree by evolutionary events other than speciations. However, not all phylogenies are trees: hybridization can occur and create new species and this results into reticulate phylogenies. Here, we consider the problem of reconciling a gene tree with a species network via duplication and loss events. Two variants are proposed and solved with effcient algorithms: the first one finds the best tree in the network with which to reconcile the gene tree, and the second one finds the best reconciliation between the gene tree and the whole network.

## Background

Reconciliations explain topology incompatibilities between a species tree and a gene tree by evolutionary events - other than speciation - affecting genes [[1], for a review]. However, not all phylogenies are trees: indeed, hybridization can occur and create new species [2] and this results into reticulate phylogenies, i.e. species (phylogenetic) networks [3]. In [4], the authors presented a first contribution toward solving a problem similar to the reconciliation problem, namely the cophylogeny problem [5-8], on networks. In their article, they first propose a polynomial algorithm to solve this problem on dated host trees taking into account codivergence, duplication, host switching, and loss events. This model is similar to the DTL model in gene tree reconciliation - that takes into account speciation, duplication, transfer, and loss events [[9], among others]. However, when extending the cophylogeny problem to species networks, their model may not be the more pertinent one for the DTL problem. Indeed, in [4] the parasite tree that is "reconciled" with the host network can take any path in the latter, modeling the fact that some hybridization species can receive the parasites of both parents. In the problem of gene tree reconciliation, their model is more adapted to novel hybridizations, where the genes still keep trace of the polyploidy due to the hybridization. But, for ancient hybridizations, the polyploidy of the extant species being reduced, a model where each gene of an hybridization species can be inherited from at most one of its two parents is more pertinent. In other words, for solving the latter problem, we are interested in finding a tree that is "displayed" by the species network such that its reconciliation with a given gene tree is optimum. We propose an efficient algorithm that takes into account duplication and loss events whose complexity does not depend on the number of hybridization events in the species network but only on the *level *of the network, where the level is a measure of how much the network is "tangled". Moreover, we propose a faster algorithm solving the problem described in [4] when restricting to duplication and loss events (that is, host switching is not taken into account).

### Basic notions

We start by giving some basic definitions that will be useful in the paper.

**Definition 1 (Rooted phylogenetic network)***A rooted phylogenetic network N on a label set  X is a rooted directed acyclic graph with a single root where each outdegree-0 node (the leaves) is labelled by an element of  X. The root of N, denoted by r*(*N*), *has indegree *0 *and outdegree *2. *All other internal nodes have either indegree *1 *and outdegree *2 *(speciation node), or indegree *2 *and outdegree *1 *(hybridization node)*.

Denote by *V*(*N*), *I*(*N*), *E*(*N*), *L*(*N*) and L(N) respectively the set of nodes, internal nodes (nodes with outdegree greater than 0), edges, leaves and leaf labels of *N*. The size of *N*, denoted by |*N*|, is equal to |*V*(*N*)| + |*E*(*N*)|. Given *x *in *V*(*N*), we denote by *N_x _*the subnetwork of *N *rooted at *x*, i.e. the subgraph of *N *consisting of all edges and nodes reachable from *x*. If *x *is a leaf of *N*, we denote by *s*(*x*) the label of *x *in L(N). If *x *is a speciation node, we denote by *p*(*x*) the only parent of *x*.

Given two nodes *x *and *y *of *N*, we say that *x *is *lower or equal *to *y *in *N*, denoted by *x *≤_*N *_*y *(resp. *lower*, denoted by *x <_N _y*), if and only if there exists a path (possible reduced to a single node) in *N *from *y *to *x *(resp. and *x *≠ *y*). If *x *≤*_N _y*, then, for every path *p *from *y *to *x*, denote by *length*(*p*) the number of speciation nodes in *N *such that *x *<*_N _z *≤*_N _y*. If *N *is a tree, then, for every two nodes *u, v *of *N*, *LCA_N _*(*u, v*) [10] is the lowest node of *N *that is above or equal to both *u, v*.

**Figure 1:**
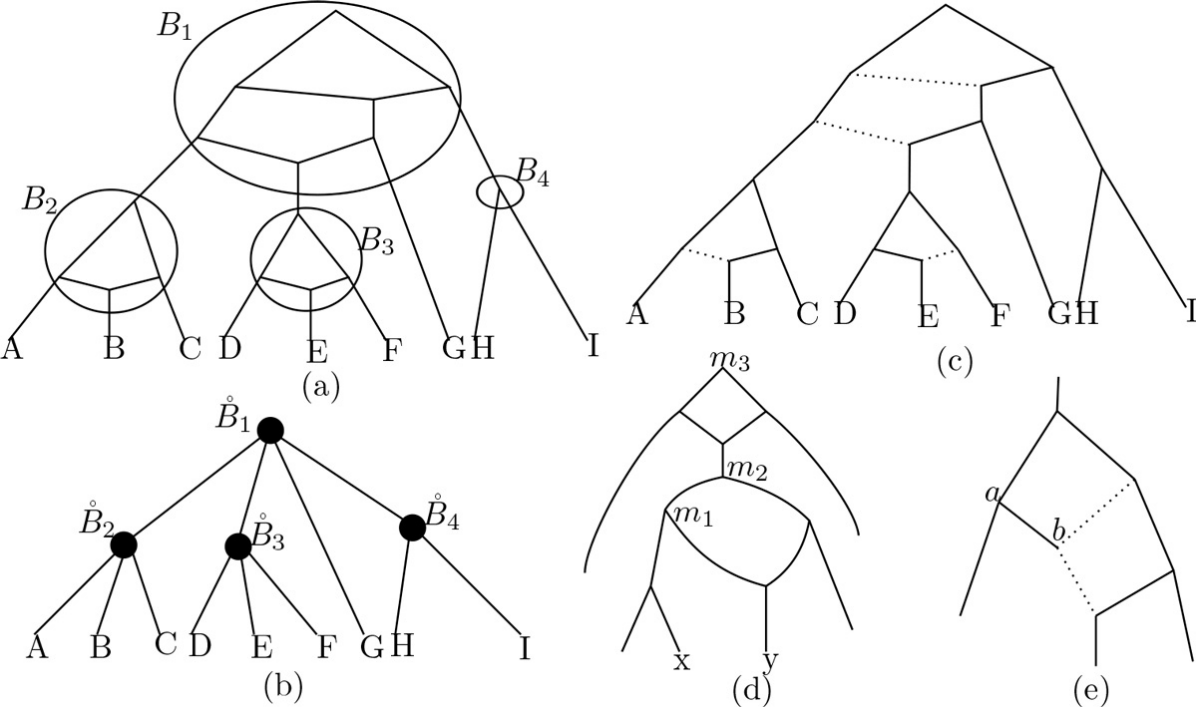
**(*a*) An example of a level-2 network *N*, (*b*) the tree *bc*(*N*), (c) a switching of *N *where the switched-off edges are dotted, (d) a network *N' *such that **$M_{N^{\prime }}\left(x,\;y\right)\;=\left\{m_{\text{1}},\;m_{\text{2}},\;m_{\text{3}}\right\}$**, (e) another switching of a network**. Note that the edge (*a*; *b*) will also be switched off since all out-going edges of *b *are off.

Given a speciation node *x *in *N*, two paths of *N *starting from *x *are said to be *separated *if each path contains a different child of *x*. Let *x, y *be two nodes of *N*. Denote by $M_{N}\left(x,y\right)$ the set of nodes *z *of *N *such that there exist two separated paths in *N *from *z *to *x *and *y*. For example, in Figure 1(d), $M_{N'}\left(x,y\right)=\left\{m_{1},m_{2},m_{3}\right\}$. Note that all nodes in $M_{N}\left(x,y\right)$ are speciation nodes and, when *N *is a tree and *x, y *are not comparable, *M_N _*(*x, y*) contains exactly one node, which coincides with *LCA_N _*(*x, y*).

If every biconnected component of *N *has at most *k *hybridization nodes, we say that *N *is of *level-k *[11]. A rooted phylogenetic tree is a rooted phylogenetic network with no hybridization nodes, i.e. a level-0 network. In the following, we will refer to rooted phylogenetic networks and rooted phylogenetic trees simply as *networks *and *trees*, respectively. In this paper, we allow trees to contain *artificial *nodes, i.e. nodes with indegree and outdegree 1.

Let *B *be a biconnected component of a network *N*. Then *B *contains exactly one node *r*(*B*) without ancestors in *B *[[12], Lemma 5.3]; we call *r*(*B*) the root of *B*. If *B *is not trivial, i.e. *B *consists of more than one node, we can *contract *it by removing all nodes of *B *other than *r*(*B*), then connect *r*(*B*) to every node with indegree 0 created by this removal. Then the following definitions are well-posed.

**Definition 2 (Tree ***bc*(*N*)**) ***Given a network N, the tree bc*(*N*) *is obtained from N by contracting all its biconnected components*.

For example, Figure 1(a, b) shows respectively a level-2 network *N *and its associated tree *bc*(*N*).

Let denote by $\overset{\circ }{B}$ the node in *bc*(*N*) that corresponds to a biconnected component *B *in *N*. Given two biconnected component *B_i_, B_j _*, we say that *B_i _*≤ *_N _B_j _*(resp. *B_i _<_N _B_j _*) if and only if $\overset{\circ }{B_{i}}\leq_{bc(N)}\overset{\circ }{B_{j}}$ (resp. $\overset{\circ }{B_{i}}{<}_{bc(N)}\overset{\circ }{B_{j}}$). We say that *B_i _*is the parent (resp. a child) of *B*_*j *_if $\overset{\circ }{B_{i}}$ is the parent (resp. a child) of $\overset{\circ }{B_{j}}$ in _bc_(*N*). We also denote by *LCA_N_*(*B_i_*, *B_j_*) the biconnected component corresponding to ${LCA}_{bc(N)}(\overset{\circ }{B_{i}},\overset{\circ }{B_{j}})$ in *N*.

**Definition 3 (Elementary network) ***Given a network N, each biconnected component B that is not a leaf of N defines an elementary network, denoted by N*(*B*), *consisting of B and all cut-edges coming out from B*.

**Definition 4 (Switchings of a network **[13]**) ***Given a network N, a switching S of N is obtained from N by choosing, for each hybridization node, an incoming edge to *switch on *and the other to *switch off. *Once this is done, we also switch off all switched-on edges with the target node having only switched-off outgoing edges (see *Figure 1(e)* for an example). For each bi-connected component B of N, we also denote by S*(*B*) *the subgraph of S restricted to N*(*B*).

Switchings will be used in the next section to model gene histories for genes evolving in a species network. For example, Figure 1 presents in (*c*) a switching of the level-2 network in (*a*). We denote by *V_on_*(*S*) the set of nodes of *S *that are not an endpoint of any switched-off edge. A path of *S *is a path of *N *that uses only switched-on edges.

Hereafter, *G *will denote a tree and *N *a network such that there is a bijection between *L*(*N*) and L(N) and L(G)⊆L(N). In the gene tree reconciliation problem, *G *represents a gene tree such that each leaf corresponds to a contemporary gene and is labeled by the species containing this gene, while *N *is a species network such that each leaf represents an extant species. In the cophylogeny problem, *G *represents a parasite tree such that each leaf corresponds to a parasite species that is labeled by the species that hosts it, while *N *is a host network such that each leaf represents an extant species.

### Reconciliations

We will now extend the definition of DL reconciliation in [14] to networks. In a DL reconciliation, each node of *G *is associated to a node of *S *and an event - a speciation (S), a duplication (D) or a contemporary event (ℂ) - under some constraints. A contemporary event  ℂ associates a leaf *u *of *G *with a leaf *x *of *S *such that *s*(*u*) = *s*(*x*). A speciation in a node *u *of *G *is constrained to the existence of two separated paths from the mapping of *u *to the mappings of its two children, while the only constraint given by a duplication event is that evolution of *G *cannot go back in time. More formally:

**Definition 5 (Reconciliation) ***Given a tree G and a network N such that *L(G)⊆L(N), *a *reconciliation *between G and N is a function α that maps each node u of G to a pair *(*α_r_*(*u*), *α*_*e*_(*u*)) *where α*_*r*_(*u*) *is a node of V*(*N*) *and α_e_*(*u*) *is an event of type * S* or* D* or* ℂ, *such that:*

• $\alpha_{e}\left(u\right)=\mathbb{C}$* if and only if u *∈ *L*(*G*), *α_r_*(*u*)∈*L*(*N*) *and s*(*u*) = *s*(*α_r_*(*u*));

• *for every u *∈ *I*(*G*) *with child nodes *{*u*_1_, *u*_2_}, *if *$\alpha_{e}\left(u\right)=S$, *then *$\alpha_{r}(u)\in {ℳ}_{N}(\alpha_{r}(u_{1}),\alpha_{r}(u_{2}))$;

• *for any two nodes u, v of V*(*G*) *such that v <_G _u, if *$\alpha_{e}\left(u\right)=D$, *then α_r _*(*v*) ≤*_N _α_r_*(*u*). *Otherwise, α_r _*(*v*) *<_N _α_r_*(*u*).

Note that, if *N *is a tree, this definition coincides with the one given in [14].

The number of duplications of *α*, denoted by *d*(*α*) is the number of nodes *u *of *G *such that $\alpha_{e}\left(u\right)=D$. Since in a network there can be several paths between two nodes, we count the number of losses on shortest paths, as done in [4]. In more details, given two nodes *x, y *of *N*, the distance between *x *and *y*, denoted by *dist*(*x, y*), is defined as follows:

• If *y *≤*_N _x*, then *dist*(*x, y*) = min*_p _length*(*p*) over all possible paths *p *from *x *to *y*;

• otherwise, *dist*(*x, y*) = +∞.

Then, for every *u *∈ *I*(*G*) with child nodes {*u*_1_, *u*_2_}, the number of losses associated with *u *in a reconciliation *α*, denoted by *l_α_*(*u*), is defined as follows:

• if $\alpha_{e}\left(u\right)=S$, then *l_α_*(*u*) = *min*{*dist*(*x*_1_, *α_r_*(*u*_1_)) + *dist*(*x*_2_, *α_r_*(*u*_2_)), *dist*(*x*_1_, *α_r_*(*u*_2_))+*dist*(*x*_2_, *α_r_*(*u*_1_))} where *x*_1_, *x*_2 _are the two children of *α_r_*(*u*);

• if $\alpha_{e}\left(u\right)=D$, then *l_α_*(*u*) = *dist*(*α_r_*(*u*), *α_r_*(*u*_1_)) + *dist*(*α_r_*(*u*), *α_r_*(*u*_2_)).

The number of losses of a reconciliation *α*, denoted by *l*(*α*), is the sum of *l_α_*(·) for all internal nodes of *G*. Thus, the cost of *α*, denoted by *cost*(*α*), is *d*(*α*)·*δ *+ *l*(*α*)·*λ*, where *δ *and *λ *are respectively the cost of a duplication and a loss event. We use *cost*(*G,N*) to denote the cost of the minimum reconciliations between *G *and *N*. A reconciliation having the minimum cost is called a *most parsimonious reconciliation*.

Let *S *be a switching of *N*, then a reconciliation between *G *and *S *is defined similarly to Definition 5 except that only switched-on edges are considered when defining paths, and only nodes in *V_on_*(*S*) are counted for calculating the length of the shortest path in the definition of *dist*. This is done to model the fact that, since each gene of an hybridization species is inherited from one of its two parents, we should not count as a loss the fact that the other parent does not contribute. Moreover, note that, for every two nodes *x, y *of *S *such that *x ≤_S _y*, there is a unique path from *y *to *x *in *S*.

When *N *is a tree, there is a unique reconciliation (the *LCA reconciliation*) between *G *and *N *which has minimum cost and this reconciliation can be found in *O*(|*G*|) time [15-18] as follows:

• for each node *u *in *L*(*G*), *α_r_*(*u*) is defined as the only node *x *of *S *such that *s*(*u*) = *s*(*x*), and $\alpha_{e}\left(u\right)=\mathbb{C}$;

• for each node *u *in *I*(*G*) with child nodes {*u*_1_, *u*_2_}, *α_r_*(*u*) = *LCA_N _*(*α_r_*(*u*_1_), *α_r_*(*u*_2_)); if *α_r_*(*u*_1_) *≤_N _α_r_*(*u*_2_) or *α_r_*(*u*_2_) *≤_N _α_r_*(*u*_1_) then $\alpha_{e}\left(u\right)=D$; otherwise $\alpha_{e}\left(u\right)=S$.

In the LCA reconciliation, the mapping *α_e _*is totally defined by *α_r_*, hence it can be omitted, and we will use *α *to refer to *α_r_*. Note that the algorithms used on trees to find the LCA reconciliation can also be used on switchings, which - when only switched-on edges are considered - are actually trees. Hereafter, when we refer to *the *reconciliation between a tree and a switching, we refer to the LCA reconciliation between them. The problems in which we are interested in here are defined as follows:

**Problem 1 **BEST SWITCHING FOR THEDL MODEL

**Input ***A tree G, a network N such that *L(G)⊆L(N), *and positive costs δ and λ for respectively * D* and * L* events*.

**Output ***A switching S of N such that the cost*(*G, S*) *is minimum over all switchings of N*.

**Problem 2 **MINIMUMDL RECONCILIATION ON NETWORKS

**Input ***A tree G, a network N such that *L(G)⊆L(N), *and positive costs δ and λ for respectively * D* and * L* events*.

**Output ***A minimum reconciliation between G and N*.

**Remark 1 ***For the sake of simplicity, we suppose that G does not contain any internal node u such that *$|L\left(G_{u}\right)|=\text{1}$* (i.e. all nodes of G_u _are mapped to a leaf of N). If it is not the case, we can simplify the instance by replacing in G each such subtree G_u _by a leaf labeled by the unique label in *$L\left(G_{u}\right)$* and compute a reconciliation for the simplified tree G'. A parsimonious reconciliation for G can be easily obtained from a parsimonious one for G'*.

## Method

### Best switching

We start by proving that finding the best switching for the DL model is NP-hard:

**Theorem 1 ***Problem 1 is NP-hard*.

*Proof: *To prove the theorem, we reduce Problem 1 to the TREE CONTAINMENT problem, which is NP-hard [19]. The TREE CONTAINMENT problem asks the following "Given a network *N *and a tree *T*, both with their leaf sets bijectively labeled by a label set  X, is there a switching *S *of *N *such that *T *can be obtained by *S *deleting all switched-off edges and nodes with indegree and outdegree 1?". Now, assume there is an algorithm  A to solve Problem 1 in polynomial time. Then, it is easy to see that, since *λ*, *δ *> 0, there is a solution of Problem 1 with cost 0 if and only if *G *is contained in *N*. Therefore, this method would provide a polynomial-time algorithm to solve the TREE CONTAINMENT problem, which is impossible.

In the following, we present a fixed-parameter tractable algorithm [20] in the level of the network to solve Problem 1. To do so, we need to introduce some notations.

**Definition 6 (Mapping ***B***) ***For every u *∈ *V*(*G*), *B*(*u*) *is defined as the lowest biconnected component B of N such that *ℒ(*N*_*r*(*B*)_) *contains *ℒ(*G_u_*).

Then the following remark holds:

**Remark 2 ***If u *∈ *L*(*G*), *then B*(*u*) *is the only leaf of N such that s*(*u*) = *s*(*B*(*u*)). *If u *∈ *L*(*G*), *then B*(*u*) = *LCA_N _*(*B*(*u*_1_), *B*(*u*_2_)) *where u*_1_, *u*_2 _*are child nodes of u in G*.

We define by *G_N _*the tree obtained from *G *by adding some artificial nodes on the edges of *G *and label each node of *G_N _*by a biconnected component of *N *via an extension of the labeling function *B*(·) as follows:

**Definition 7 (Tree ***G_N _***) ***The tree G_N _is obtained from G as follows: for each internal node u in G with child nodes u*_1_, *u*_2 _*such that there exist k biconnected components ${B_{i}}_{{}_{\text{1}}}{>}_{N}..\;.{>}_{N}B_{i_{k}}$strictly below B*(*u*) *and strictly above B*(*u*_1_), *we add k artificial nodes v*_1 _> ... >*v_k _on the edge *(*u, u*_1_), *and B*(*v_j _*) *is fixed to $B_{i_{j}}$. We do the same for u*_2_.

**Figure 2:**
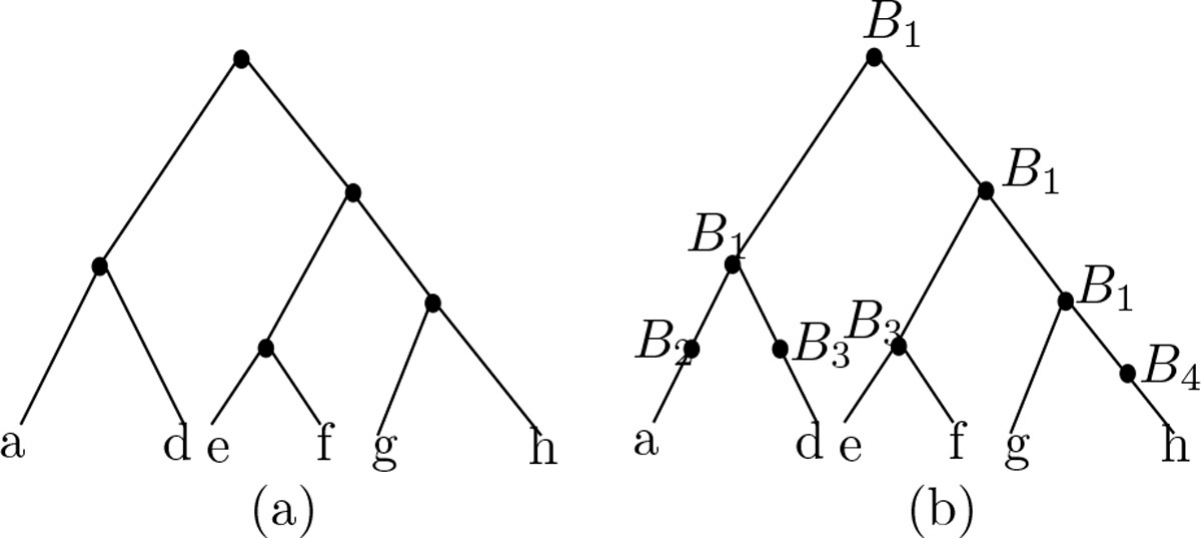
**(a) A gene tree *G *and (b) the tree *G_N _*along with the labeling *B*(·) of its nodes (*N *here is the network in **Figure 1(a)**)**.

See Figure 2 for an example of *G_N _*. We have the following lemma:

**Lemma 1 ***Let u be a node of I*(*G_N _*), *and u' be one of the children of u. If u is an artificial node, then B*(*u*) *is the parent of B*(*u'*) *and a child of B*(*p*(*u*))*; otherwise, B*(*u'*) *is either equal to B*(*u*) *or one of its children*.

*Proof: *Suppose that *u *is an artificial node. Then, by Definition 7, *B*(*p*(*u*)), *B*(*u*), *B*(*u'*) are three consecutive nodes of *G_N _*, thus *B*(*u*) is the parent of *B*(*u'*) and a child of *B*(*p*(*u*)). Suppose now that *u *is not an artificial node, and let *u" *be the child of *u *in *G *such that *u" *≤*_Gn _u' *≤*_Gn _u*. If *B*(*u"*) = *B*(*u'*), then by definition, *u" *= *u' *because no artificial node is added between *u *and *u"*, and thus the claim holds. If *B*(*u'*) ≠ *B*(*u"*), then by Definition 7, *B*(*u'*) is the highest biconnected component of *N *that is below *B*(*u*) and above *B*(*u"*), which is then a child of *B*(*u*).

**Definition 8 **(*G_B _*) *Let B be a biconnected component of N different from a leaf, then G_B _is the set of all maximal connected subgraph H of G_N _such that B*(*u*) = *B for every u *∈ *I*(*H*).

**Figure 3:**
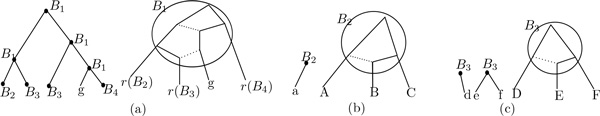
**An example of execution of Algorithm 1 on the tree *G *in **Figure 2(a)** and the network *N *in **Figure 1(a). (a) $G_{B_{1}}$ and a switching of *N*(*B*_1_) on which its reconciliation with $G_{B_{1}}$ contains 1 duplication and 2 losses. (b) $G_{B_{2}}$ and a switching of *N*(*B*_2_) on which the reconciliation with $G_{B_{2}}$ contains one loss. (c) $G_{B_{3}}$ and a switching of *N*(*B*_3_) on which the reconciliation with $G_{B_{3}}$ contains 2 losses.

For example, in Figure 3, ${G_{B}}_{{}_{\text{1}}}$ consists of one binary tree, ${G_{B}}_{{}_{\text{2}}}$ consists of one edge, and ${G_{B}}_{{}_{\text{3}}}$ contains 1 tree and 1 edge.

**Lemma 2 ***Let B be a biconnected component of N different from a leaf, then we have the following:*

**(i) ***for every H *∈ *G_B_, H is either a binary tree or an edge whose upper extremity is an artificial node. Moreover, for every leaf u of H, B*(*u*) *is a child of B*.

**(ii) ***if B *= *B*(*r*(*G*)), *then G_B _consists of one binary tree*.

*Proof: ***(i) **First, suppose that *I*(*H*) contains an artificial node *u*. Then this artificial node is the only internal node of *H*; indeed, by Lemma 1, the value of *B*(·) for the parent and the child of *u *are both different from *B*. Thus, *H *consists of only one edge whose upper extremity is *u*. If *I*(*H*) does not contain any artificial node, it follows that *H *is a binary tree. Moreover, by Lemma 1 and Definition 8, *B*(*u*) is a child of *B *for every leaf *u *of *H*.

**(ii) **Suppose now that *B *= *B*(*r*(*G*)), and *G_B _*contains at least two components *H*_1_, *H*_2_, rooted at two different nodes *r*_1_, *r*_2 _where *B*(*r*_1_) = *B*(*r*_2_) = *B*. Let $r=LCA_{G_{N}}(r_{1},r_{2})$, then, by Definition 6, *B*(*v*) = *B *for every node *v *on the two paths from *r *to *r*_1 _and to *r*_2_, because *B*(*r*(*G*)) = *B*(*r*_1_) = *B*(*r*_2_) = *B *and $r_{\text{1}},\;r_{\text{2}}\leq_{G_{N}}r\;\leq_{G_{N}}r\left(G\right)$. But this contradicts the maximality of *H*_1 _and *H*_2 _since they can both be extended. Hence *G_B _*contains only one component. Suppose that this component is an edge; thus, its upper extremity is an artificial node that has been added on the path from a node *u *to a node *v *of *G *where *u *is strictly higher than *r*(*G*). But this is not possible, because *r*(*G*) is the highest node of *G*. Therefore, *G_B _*contains one binary tree.

Given a biconnected component *B_i _*different from a leaf, denote by $G_{B_{i}}^{t}$ the set of binary trees of $G_{B_{i}}$, and $G_{B_{i}}^{e}$ the set of edges of $G_{B_{i}}$. Let *S_i _*be a switching of *N*(*B_i_*), and let *H *be a tree in $G_{B_{i}}^{t}$. By Lemma 2, for every *u *∈ *L*(*H*), *B*(*u*) is a child of *B_i _*and thus *r*(*B*(*u*)) is a leaf of *N*(*B_i_*), which is also a leaf of *S_i_*. Hence, we can define a reconciliation between *H *and *S_i_*, denoted by $\beta_{S_{i}}^{H}$, such that each leaf *u *of *H *is mapped to the leaf *r*(*B*(*u*)) of *S_i_*.

**Lemma 3 ***Let S be a switching of N, and let α be the reconciliation between G and S. For every u *∈ *I*(*G*), *there exists *$H\in G_{B\left(u\right)}^{t}$*such that u *∈ *I*(*H*), *and *$\alpha (u)=\beta_{S(B(u))}^{H}(u)$.

The proof of this lemma is deferred to the appendix. The following definition will be useful later on.

**Definition 9 (***cost*(*H,S_i_*)**) ***Let B_i _be a biconnected component of N different from a leaf, and S_i _a switching of N*(*B_i_*)*; then cost*(*H,S_i_*) *is defined as follows:*

• $\forall H\in G_{B_{i}}^{t}$, $cost\left(H,S_{i}\right)=cost\left(\beta_{S_{i}}^{H}\right)$*if B_i _*= *B*(*r*(*G*)), *and *$cost(H,S_{i})=cost(\beta_{S_{i}}^{H})+\lambda \;\cdot dist(r\left(S_{i}\right),\beta_{S_{i}}^{H}\left(r\left(H\right)\right)$*otherwise;*

• $\forall H\in G_{B_{i}}^{e}$, $cost(H,S_{i})=\lambda \;\cdot dist(r\left(S_{i}\right),\beta_{S_{i}}^{H}\left(r\left(B(u)\right)\right)$*where u is the only leaf of H*.

As we will see later, this cost corresponds to the contribution of *H *to a reconciliation between *G *and any switching of *N *that contains *S_i_*. For example, in Figure 3(b), *H *is the edge (*B*_2_, *a*) and, if *S_i _*is the switching on the left, then *cost*(*H,S_i_*) = *λ*.

The next lemma is a central one, and it permits to solve Problem 1 independently per each biconnected component of *N *:

**Lemma 4 ***Let {B*_1_,..., *B_p_} be the biconnected components of N that are not leaf nodes, and let S be a switching of N where each elementary network N(B_i_) has S_i _as switching. Then *$cost\left(G,S\right)=\overset{p}{\underset{i=1}{\sum }}\sum_{H\in G_{B_{i}}}cost\left(H,S_{i}\right).$

*Proof: *Let *α *be the reconciliation between *G *and *S*. Denote by *d_α_*(*S_i_*) the number of nodes in *I*(*S_i_*) associated to a duplication by *α*. By Remark 1, no duplication happens at leaves of *S*. Additionally, $\cup_{B_{i}}\cup_{H\in G_{B_{i}}^{t}}I\left(H\right)=I\left(G\right)$ and the sets of internal nodes of $G_{B_{i}}^{t}$ are disjoint. Hence, we have $d(\alpha )\;=\sum_{i=1}^{i}d_{\alpha }(s_{i})=\sum_{i=1}^{p}\sum_{H\in G_{B_{i}}^{t}}d(\beta_{S_{i}}^{H})$ because $\beta_{S_{i}}^{H}\left(u\right)=\alpha \left(u\right)$ for every internal node *u *of *H *(Lemma 3).

Let us now consider the loss count. Note that a node/edge is on (resp. off) in *S_i _*if and only if it is also on (resp. off) in *S*. Let *x, y *be two nodes of *S *such that *y ≤_S _x*. Then we define $dist_{S_{i}}(x,y)$ as the number of nodes *z *in *V_on_*(*S_i_*) \ *L*(*S_i_*) such that *y <_S _z ≤_S _x*. Then, for each internal node *u *of *G*, we define the number of losses associated with *u *in *S_i_*, denoted by *l_α_*(*u, S_i_*), similarly to *l_α_*(*u*) but using the function $dist_{S_{i}}$ instead of *dist*. Then, $l_{\alpha }\left(u\right)=\sum_{i=1}^{p}l_{\alpha }(u,S_{i})$. Now, let *u*_1_, *u*_2 _be two children of *u *in *G*, then *l_α_*(*u, S_i_*) *>*0 only if the path from *α*(*u*) to either *α*(*u*_1_) or *α*(*u*_2_) in *S *contains at least a node of *B_i_*. Therefore, we have three possible cases: (1) *α*(*u*) is in is in *B_i_*, (3) *α*(*u*) is above *r*(*B_i_*) while either *α*(*u*_1_) or *α*(*u*_2_) is in a biconnected component below *B_i_*. In case (1), by Lemma 3, there exists a binary tree *H *of $G_{B_{i}}^{t}$ such that *u*∈*I*(*H*), and $\alpha \left(v\right)\;=\beta_{S_{i}}^{H}\left(v\right)$ for every *v*∈*I*(*S_i_*), thus $l_{\alpha }\left(u,\;S_{i}\right)\;=l_{\beta_{s_{i}}^{H}}\left(u\right)$. Now, let suppose *S_i _*that case (2) holds for *u*_1_, then *u*_1 _must be the root of a binary tree *H*_1 _of *G^t ^*, and the contribution of *u*_1 _to *l_α_*(*u, S_i_*) is $dist\left(r\left(S_{i}\right),\beta_{S_{i}}^{H_{1}}\left(r\left(H_{\text{1}}\right)\right)\right)$. Note that in this case, *u*_1 _≠ *r*(*G*). Finally, let suppose that case (3) holds for *u*_1 _and let *u_a_, u_b _*be the artificial nodes added on the edge (*u, u*_1_) of *G *such that *B*(*u_a_*) = *B_i _*and *B*(*u_b_*) is a child of *B_i_*. Hence, $\left(u_{a},\;u_{b}\right)\in G_{B_{i}}^{e}$, and the contribution of *u*_1 _to *l_α_*(*u, S_i_*) is *dist*(*r*(*S_i_*), *r*(*B*(*u_b_*))). Let call $V_{i}^{1},V_{i}^{2},V_{i}^{3}$ the set of nodes *u *in the first, second, and third case. By construction, *V*^1 ^is disjoint from $V_{i}^{2}$ and $V_{i}^{3}$. Moreover, $V_{i}^{2}$ and $V_{i}^{3}$ are disjoint because if a node *u *has two children *u*_1_, *u*_2 _such that *u*_1 _is in *B_i_*, and *u*_2 _is below *B_i_*, then *u *must be in *B_i_*, i.e. cannot be above *r*(*B_i_*). Thus,

$$l\left(\alpha \right)=\underset{u\in I\left(G\right)}{\sum }l_{\alpha }\left(u\right)=\underset{u\in I\left(G\right)}{\sum }\overset{p}{\underset{i=1}{\sum }}l_{\alpha }\left(u,S_{i}\right)=\overset{p}{\underset{i=1}{\sum }}\underset{u\in V_{i}^{1}\cup V_{i}^{2}\cup V_{i}^{3}}{\sum }l_{\alpha }(u,S_{i}).$$

Therefore,

$$\begin{array}{l} cost(G,S)=\delta \cdot \;d(\alpha )+\lambda \;\cdot l(\alpha )=\sum_{i=1}^{p}(\delta \cdot \;d_{\alpha }(S_{i})+\\ \lambda \cdot \sum_{u\in I(G)}l_{\alpha }(u,S_{i}))=\sum_{i=1}^{p}(\delta \cdot \;\sum_{H\in G_{B_{i}}^{t}}d(\beta_{i}^{H})+\\ \lambda \;\cdot \sum_{u\in V_{i}^{1}\cup V_{i}^{2}\cup V_{i}^{3}}l_{\alpha }(u,S_{i})=\sum_{i=1}^{p}([\lambda \cdot \;\sum_{u\in V_{i}^{3}}\left(u,S_{i}\right)]+\\ +[\delta \cdot \sum_{H\in G_{B_{i}}^{t}}d(\beta_{S_{i}}^{H})+\lambda \;\cdot \sum_{u\in V_{i}^{1}}l_{\alpha }(u,S_{i})+\\ \lambda \;\cdot \sum_{u\in V_{i}^{2}}l_{\alpha }(u,S_{i})]). \end{array}$$

As proved above (in case (3)), the first term between squared brackets is equal to $\sum_{H\in G_{B_{i}}^{t}}cost\left(H,S_{I}\right)$ by Definition 9. In the second term between squared brackets, the sum of the two first factors is exactly $\sum_{H\in G_{B_{i}}^{t}}cost\left(\beta_{S_{i}}^{H}\right)$ (as proved in case (1)), while the last factor is equal to $\lambda \cdot \sum_{H\in G_{B_{i}}^{t}}dist(r(S_{i}),\beta_{S_{i}}^{H}(r(H))$ (as proved in case (2)). Note that as mentioned in case (2), only nodes that are not the root of *G *are considered. Hence, the second term between squared brackets corresponds to $\sum_{H\in G_{B_{i}}^{t}}cost\left(H,S_{i}\right)$ by Definition 9.

Therefore, $cost\left(G,S\right)\;=\sum_{i=1}^{p}\sum_{H\in G_{B_{i}}}cost(H,S_{i})$ and this concludes the proof.

Algorithm 1 computes the switching of *N *for which its reconciliation with *G *has the smallest cost, by analyzing each biconnected component of *N *independently. See Figure 3 for an example of application of this algorithm to the species network in Figure 1(a) and the gene tree in Figure 2(a).

**Algorithm 1 **Solving Problem 1

1: **Input: **A species network *N *and a gene tree *G *such that L(G)⊆L(N), and positive costs *δ*, *λ *for duplication and loss events, respectively.

2: **Output: **A switching *S *of *N *that is optimal in the sense of Problem 1.

3: Compute the tree *G_N _*and its labeling function *B*(·);

4: Compute $G_{B_{i}}$ for each biconnected component *B_i _*of *N *that is not a leaf;

5: **for **each biconnected component *B_i _*of *N ***do**

6:      **for **each switching $S_{i}^{j}$ of *N*(*B_i_*) **do**

7:         $cost_{j}=\sum_{H\in G_{B_{i}}}cost\left(H,S_{i}^{j}\right)$;

8:     $S_{i}^{m}\leftarrow$ the switching of *N*(*B_i_*) with the lowest value of *cost_j _*over all *j*;

9: Return the switching *S *of *N *in which each elementary network *N*(*B_i_*) has $S_{i}^{m}$ as switching.

**Theorem 2 ***Let N be a level-k network with p biconnected components. Algorithm 1 runs in O*(|*N*| + 2^*k*^·*p*·|*G*|) *time and returns a switching S of N such that cost*(*G,S*) *is minimum*.

*Proof: ***Complexity: **It is well-known that all biconnected components of *N *can be computed in linear time, i.e. *O*(|*N*|), by using depth-first-search [10]. After a linear preprocessing, *LCA *operations on a tree can be performed in constant time [21,22]. Thus, from Remark 2, the mapping *B*(·) can be computed in *O*(|*G_N_*|). Hence, the tree *G_N _*can be constructed in times *O*(|*G_N _| *+ |*N*|).

By a simple traversal of *G_N _*which takes time *O*(|*G_N _*|), we can compute $G_{B_{i}}$ for all *B_i_*. Each biconnected component *B_i _*of *N *has at most *k *hybridization nodes, then *N*(*B_i_*) has at most 2*^k ^*switchings. For each switching $S_{i}^{j}$ of *N*(*B_i_*), it takes *O*(|*G_B _*|) to compute *cost_j _*[23,24]. The overall size of all *G_Bi _*is the size of *G_N _*. Therefore, the total complexity of the loop at lines 5 - 8 is *O*(2*^k ^*·|*G_N _*|). Each edge of *G *can have at most *p *artificial nodes added to it. Hence in the worst case *O*(|*G_N _*|) = *O*(|*G*|·*p*), i.e. the total complexity of Algorithm 1 is *O*(|*N*| + 2*^k^*·*p*·|*G*|).

**Correctness: **Let *S *be a switching of *N *where each elementary network *N*(*B_i_*) has *S_i _*as the switching. By Lemma 4, *cost*(*G,S*) = $\sum_{i=1}^{p}\sum_{H\in G_{B_{i}}}cost\left(H,S_{i}\right)$. Hence *cost*(*G,S*) is minimum if and only if $\sum_{H\in G_{B_{i}}}cost\left(H,S_{i}\right)$ is minimum for every *S_i_*. Lines 5-8 in Algorithm 1 search, for each network *N*(*B_i_*), the tree $S_{i}^{m}$ such that $\sum_{H\in G_{B_{i}}}cost\left(H,S_{i}\right)$ is minimum, which implies the correctness of the algorithm.

### Minimum Reconciliation on Networks

Given a tree *G *and a network *N*, in [4] the authors prove that reconciling *G *on *N *can be solved in polynomial time, when host switchings (or transfer events, in the DTL reconciliation terminology) are also accounted for. In their model, each node of *N *is dated by a single date while each node of *G *is dated by a set of dates, and they search for a parsimonious reconciliation between *G *and *N*, i.e. one that has minimum cost, under the constraint that an event associated to a node *u *of *G *can only happen at a node/edge of *N *whose date is contained in the set of possible dates of *u*. Although the algorithm complexity stays polynomial, it is very high: *O*(*τ*^3^·|*G*|·|*N*|^5^) for a binary tree and a binary network, where *τ *is the number of possible dates of the nodes of *G *and *N*, which is at most *O*(|*G*| + |*N*|). A drawback of this model is that it requires information on the dates that is often not available. Moreover, transfers are not always possible in all parts of Tree of Life. Here, we take into account only speciation, duplication and loss events, and consider *G *and *N *as undated (see Problem 2 and Definition 5 for more details). Using a similar dynamic algorithm on this simpler model, and by some further analyses, we provide an algorithm that is a generalization of the LCA algorithm to networks that has a much smaller complexity than the algorithm in [4], namely *O*(*h*^2^·|*G*|·|*N*|), where *h *is the number of hybridization nodes of *N*.

Let *x, y *be two nodes of *N*. Denote by $Min_{N}\left(x,\;y\right)$ the subset of $M_{N}\left(x,\;y\right)$ such that, for every $z\in Min_{N}\left(x,\;y\right)$, there does not exist any $z^{\prime }\in Min_{N}\left(x,\;y\right)$ such that every path from *z *to *x *and to *y *passes through *z'*. For example, in Figure 1(d), *m*_1_, *m*_2_, *m*_3 _are in $M_{N}\left(x,\;y\right)$ but only *m*_1 _and *m*_2 _are in $M_{N}\left(x,\;y\right)$ because every path from *m*_3 _to *x, y *must pass *m*_2_.

For the sake of simplicity, we consider only reconciliations without duplications on hybridization nodes: indeed, since losses are not counted at hybridization nodes, a duplication on such nodes can be moved to its only child without changing the total cost of the reconciliation.

**Lemma 5 ***Let α be a reconciliation of minimum cost between G and N, then for every node u of G that has two children u*_1_, *u*_2_, *we have:*

*(i) if *$\alpha_{e}\left(u\right)=S$, * then *$\alpha_{r}\left(u\right)\in Min_{N}\left(\alpha_{r}\left(u_{\text{1}}\right),\alpha_{r}\left(u_{\text{2}}\right)\right)$;

*(ii) if *$\alpha_{e}\left(u\right)=D$, *then either α_r _*(*u*_1_) ≤_*N *_*α_r_*(*u*_2_) *and α_r_*(*u*) = *α_r_*(*u*_2_), *or α_r_*(*u*_2_) ≤_*N *_*α_r_*(*u*_1_) *and α_r_*(*u*) = *α_r_*(*u*_1_).

*Proof: *(i) Suppose that $\alpha_{e}\left(u\right)=S$, then by definition *α_r_*(*u*) must be a node of $M_{N}\left(\alpha_{r}\left(u_{\text{1}}\right),\alpha_{r}\left(u_{\text{2}}\right)\right)$. Let *x*_1_, *x*_2 _be two children of *α_r_*(*u*) such that *l_α_*(*u*) = *dist*(*x*_1_, *α_r_*(*u*_1_))+*dist*(*x*_2_, *α_r_*(*u*_2_)). Suppose that $\alpha_{r}\left(u\right)\notin Min_{N}\left(\alpha_{r}\left(u_{\text{1}}\right),\alpha_{r}\left(u_{\text{2}}\right)\right)$, then there exists a node *y *in $Min_{N}\left(\alpha_{r}\left(u_{\text{1}}\right),\alpha_{r}\left(u_{\text{2}}\right)\right)$ such that every path from *α_r_*(*u*) to *α_r_*(*u*_1_) (resp. to *α_r_*(*u*_2_)) passes for *y*. Let *y*_1_, *y*_2 _be the two children of *y*.

First, we suppose that the shortest path from *x*_1 _to *α_r_*(*u*_1_) passes through *y*, *y*_1_, while the one from *x*_2 _to *α_r_*(*u*_2_) passes *y*, *y*_2_. Consider the reconciliation *α' *such that $\alpha_{r}^{\prime }\left(v\right)=\alpha_{r}\left(v\right)$ and $\alpha_{r}^{\prime }\left(e\right)=\alpha_{r}\left(e\right)$ for every *v *≠ *u*, while $\alpha_{r}^{\prime }\left(u\right)=y$ and $\alpha_{e}^{\prime }\left(u\right)=S$. It is easy to see that *α' *respects Definition 5, and that *d*(*α*) = *d*(*α'*). Denote by *f *= *dist*(*α_r_*(*u*), *y*), then *f >*0. The numbers of losses in *α *and *α'* are different on those associated with *u *and *p*(*u*) (if *u *is not the root of *G*). We thus have *l_α_*(*p*(*u*)) ≥ *l_α'_*(*p*(*u*)) - *f *if *u *≠ *r*(*G*). Moreover, *l_α_*(*u*) = *dist*(*x*_1_, *α_r_*(*u*_1_)) + *dist*(*x*_2_, *α_r_*(*u*_2_)) = *dist*(*x*_1_, *y*) + 1 + *dist*(*y*_1_, *α_r_*(*u*_1_)) + *dist*(*x*_2_, *y*) + 1 +*dist*(*y*_2_, *α_r_*(*u*_2_)) ≥ *dist*(*α_r_*(*u*), *y*) + *dist*(*y*_1_, *α' *(*u*_1_) + *dist*(*α_r_*(*u*), *y*)+*dist*(*y*_2_, *α*' (*u*_2_))) ≥ 2·*f *+*l_α' _*(*u*). Hence, whether *u *coincides with *r*(*G*) or not, *l*(*α*) *> l*(*α'*), i.e. *cost*(*α*) *> cost*(*α'*), a contradiction.

Suppose now that both the shortest paths from *x*_1 _to *α_r_*(*u*_1_) and to *α_r_*(*u*_2_) pass through *y*, and then pass through *y*_1 _(or *y*_2_). This means that *y*_1 _is above both *α_r_*(*u*_1_) and *α_r_*(*u*_2_). Let *y' *be one of the lowest nodes below or equal to *y*_1 _that is above both *α_r_*(*u*_1_) and *α_r_*(*u*_2_). Let $y_{1}^{\prime }$, $y_{2}^{\prime }$ be the two children of *y'*, and sup-1 2 pose, without loss of generality, that $y_{1}^{\prime }$ is above or equal to *α_r_*(*u*_1_) and $y_{2}^{\prime }$ is above or equal to *α_r_*(*u*_2_). Hence, the shortest path from *x*_1 _to *α_r_*(*u*_1_) must pass, in the order, through *y*, *y*_1_, *y'*, and *y'*, while the shortest path from *x*_2 _to *α_r_*(*u*_2_) must pass through *y*, *y*_1_, *y'*, and $y_{1}^{\prime }$. By defining the reconciliation *α'*as done above apart for *α'*(*u*), which is fixed to *y'*, we arrive at a contradiction by a similar argument.

(ii) Suppose that $\alpha_{e}\left(u\right)=D$, and *α_r_*(*u*_1_), *α_r_*(*u*_2_) are not comparable. Hence, $Min_{N}\left(\alpha_{r}\left(u_{\text{1}}\right),\alpha_{r}\left(u_{\text{2}}\right)\right)$ is not empty. If $\alpha_{r}\left(u\right)\notin Min_{N}\left(\alpha_{r}\left(u_{\text{1}}\right),\alpha_{r}\left(u_{\text{2}}\right)\right)$, then there exists a node $y\in Min_{N}\left(\alpha_{r}\left(u_{\text{1}}\right),\alpha_{r}\left(u_{\text{2}}\right)\right)$ such that every path from *α_r_*(*u*) to *α_r_*(*u*_1_) and to *α_r_*(*u*_2_) must pass through *y*. Similarly to case (i), we have that the reconciliation *α' *that coincides with *α *apart for the fact that $\alpha_{r}^{\prime }\left(u\right)=y$ and $\alpha_{e}^{\prime }\left(u\right)=S$ has smaller cost than *α*, a contradiction. Hence, *α_r_*(*u*) *2 ℳin_N _*(*α_r_*(*u*_1_), *α_r_*(*u*_2_)). Considering now the reconciliation *α' *that coincides with *α *but for *α' *(*u*), which is fixed to  S. Hence *d*(*α*) = *d*(*α'*)+1, and $l_{\alpha }\left(p\left(u\right)\right)\;=l_{\alpha^{\prime }}\left(p\left(u\right)\right)$ if *u ≠ r*(*G*). Let *x*_1_, *x*_2 _be the two children of *α_r_*(*u*). If the shortest path from *α_r_*(*u*) to *α_r_*(*u*_1_) passes through *x*_1 _while the shortest path from *α_r_*(*u*) to *α_r_*(*u*_2_) passes through *x*_2_, then $l_{\alpha }\left(u\right)=\text{2}+l_{\alpha^{\prime }}\left(u\right)$. Therefore, *cost*(*α*) *> cost*(*α'*), a contradiction. Thus, both the two shortest paths from *α_r_*(*u*) to *α_r_*(*u*_1_) and *α_r_*(*u*_2_) must pass through *x*_1 _(or *x*_2_). Let *y *be one of the lowest nodes located on both of these paths. Then, *y *∈ *ℳin_N _*(*α_r_*(*u*_1_), *α_r_*(*u*_2_)). By the same argument as in the previous case, the reconciliation *α" *coinciding with *α *but for $\alpha_{r}^{\prime \prime }\left(u\right)=y$ and $\alpha_{e}^{\prime \prime }\left(u\right)=S$ must have smaller cost than *α*, a contradiction.

Hence, either *α_r_*(*u*_1_) *≤_N _α_r _*(*u*_2_) or *α_r_*(*u*_2_) *≤_N _α_r _*(*u*_1_). Suppose that the first case holds (the second case is similar), but *α_r_*(*u*) ≠ *α_r_*(*u*_2_), i.e. *α_r_*(*u*_2_) *<_N _α_r _*(*u*). Let *x*_1_, *x*_2 _be two children of *α_r_*(*u*). If the shortest path from *α_r_*(*u*) to *α_r_*(*u*_1_) passes through *x*_1 _while the shortest path from *α_r_*(*u*) to *α_r_*(*u*_2_) passes through *x*_2_, then by replacing *α_e_*(*u*) by an  S event, we obtain a reconciliation with a smaller cost. Thus, both shortest paths from *α_r_*(*u*) to *α_r_*(*u*_1_) and *α_r_*(*u*_2_) must pass through *x*_1 _(or *x*_2_). Let *y *be a node that is located on both paths such that there is no other node below *y *on these two paths. Then, *y *≠ *ℳin_N _*(*α_r_*(*u*_1_), *α_r_*(*u*_2_)). By the same argument as in the case (i), the reconciliation *α' *such that $\alpha_{r}^{\prime }\left(v\right)=\alpha_{r}\left(v\right)$, $\alpha_{e}^{\prime }\left(v\right)=\alpha_{e}\left(v\right)$ for every *v *≠ *u*, $\alpha_{r}^{\prime }\left(u\right)=y$, $\alpha_{e}^{\prime }\left(u\right)=S$ must have smaller cost than *α*, a contradiction.

Now, we are ready to describe a dynamic algorithm to compute a reconciliation of minimum cost between *G *and *N*. Let *α *be a reconciliation between *G *and *N*. For every *u *∈ *V*(*G*), denote by *cost_α_*(*u*) the cost of the reconciliation of *α *restricted to *G_u_*. Hence, if *α *is a most parsimonious reconciliation, then *cost_α_*(*u*) is the minimum cost among all reconciliations between *G_u _*and *N *that maps *u *to *α_r_*(*u*). Algorithm 2 aims at computing, for each *u*, the set  C(*u*) containing all pairs (*x,c*) such that *c *is the minimum cost among all reconciliations between *G_u _*and *N *mapping *u *to *x*. It is straightforward to see that the cost of a most parsimonious reconciliation between *G *and *N *is the minimum cost involved in a pair in  C(*r*(*G*)).

The function *merge*(*L*_1_, *L*_2_) used in Algorithm 2 takes as input two lists of pairs (*x,c*) - where *x *is a node of *N *and *c *is a real number - and merges them keeping, for each *x*, the pair (*x,c*) with the smallest value of *c*. The method *computeMin*(*y, z*) used in Algorithm 2 is detailed in Algorithm 3. This method computes, for two nodes *y, z *of *N*, the set $Min_{N}\left(y,\;z\right)$ by using two breath-first-searches (BFS) starting respectively from *y *and *z *up to the root of *N *(note that, to perform the breath-first-searches, the edges are considered as directed in the inverse order). For this, it labels each node *v *in such a way that, if *v *is not strictly above *y *and *z*, then *label *(*v*) = ∅. Otherwise, *label*(*v*) is the lowest node such that every path from *v *to *y *and *z *passes through it. This method also computes the value of the function *dist *between *y *(resp. *z*) and each node visited in the corresponding breath-first-search.

**Algorithm 2 **Solving Problem 2

1: **Input: **A network *N *and a tree *G *such that L(G)⊆L(N), and positive costs *δ*, *λ *for duplication and loss events, respectively.

2: **Output: **The set  C(*u*) of pairs (*x,c*) for every $u\in V\left(G\right)$.

3: **for **each node *u *of *G *in post-order **do**

4:    C(*u*) ← ∅;

5:   **if ***u *is a leaf **then**

6:      Let *x *be the leaf of *S *such that *s*(*x*) = *s*(*u*);

7:       C(*u*) ← {(*x*, 0)};

8:   **else**

9:      Let *u*_1_, *u*_2 _be the two children of *u*;

10:      **for **each (*y*, *c*_1_) ∈  C(*u*_1_) and each (*z*, *c*_2_) ∈  C(*u*_2_) **do**

11:         *computeMin*(*y, z*);

12:         *C *← ∅;

13:         **for **each $x\in Min_{N}\left(y,\;z\right)$**do**

14:            Let *x*_1_, *x*_2 _be the two children of *x *in *N *;

15:            *c *= *c*_1 _+ *c*_2 _+ λ·*min*{*dist*(*x*_1_, *y*) + *dist*(*x*_2_, *z*), *dist*(*x*_2_, *z*) + *dist*(*x*_1_, *y*)};

16:            *C *← *C *∪ {(*x*, *c*)};

17:         **if ***y ≤_N _z ***then**

18:            *c *= *δ *+ *λ*·*dist*(*z, y*)+ *c*_1 _+ *c*_2_;

19:            *C *← *C *∪ {(*z*, *c*)};

20:         **else if ***z ≤_N _y ***then**

21:            *c *= *δ *+ *λ*·*dist*(*y, z*) + *c*_1 _+ *c*_2_;

22:            *C *← *C *∪ {(*y*, *c*)};

23:        C(*u*) = *merge*(C(u),C);

24: Return  C.

The following theorem proves the correctness of Algorithm 2:

**Theorem 3 ***Algorithm 2 returns a matrix * C* such that, for every u *∈ *V*(*G*), (*x*,*c*) *is contained in * C(*u*) *if and only if there exists a most parsimonious reconciliation between G_u _and N mapping u to x with cost c*.

**Algorithm 3 ***computeMin*(*y, z*)

1: Proceed a BFS from *y*, store the ordered list of visited nodes in *BFS*(*y*) and compute, for each *u *in *BFS*(*y*), *dist*(*y,v*);

2: Do the same from *z*;

3: **for **each node *v *∈ *BFS*(*y*) **do**

4:   **if ***v *= *y ***or ***v *= *z ***or ***v *is not in *BFS*(*z*) **then ***label*(*v*) = ∅

5:   **else if ***v *has only one child *v*_1 _that is in *BFS*(*z*) or *BFS*(*y*) **then**

6:      *label*(*v*) = *label*(*v*_1_);

7:   **else**

8:      Let *v*_1_, *v*_2 _be the two children of *v*;

9:      **if ***label*(*v*_1_) = *label*(*v*_2_) ≠ ∅ **then**

10:         *label*(*v*) = *label*(*v*_1_);

11:      **else**

12:         *label*(*v*) = {*v*}; Add *v *into $Min_{N}\left(y,\;z\right)$;

*Proof: *For each *u *∈ *V*(*G*), we need to prove that, if *α' *is a reconciliation of minimum cost between *G_u _*on *N*, then (*α'*(*u*), *cost_α'_*) is contained in  C(*u*). This is obviously true for every leaf of *G *(by lines 5-7). Let now *u *be an internal node having two children *u*_1_, *u*_2_. Then, following Lemma 5,  C(*u*) can be computed from  C(*u*_1_) and  C(*u*_2_) by using the information contained in $Min_{N}$ and *dist*. Lines 10-23 in Algorithm 2 computes  C(*u*) following this lemma.

It remains now to prove that Algorithm 3 correctly computes $Min_{N}\left(y,\;z\right)$. For every node *v *that is above *y, z*, denote by *low*(*v*) the lowest node such that every path from *v *to *y, z *must pass through this node. There is only one such node. Indeed, suppose that there are two such nodes *m, m'*, then every path from *v *to *y *must pass through both *m, m'*, i.e. either *m *<*_N _m' *or *m' *<*_N _m*, contradicting the lowest property of *m*, *m'*. To prove the claim, we need to show that if *v *is a node of *BFS*(*y*), then:

(i) if *v *is not strictly above *y *and *z*, then *label*(*v*) = ∅;

(ii) otherwise, *label*(*v*) = *low*(*v*) and line 12 of Algorithm 3 is performed if and only if *v *is actually in $Min_{N}\left(y,\;z\right)$.

Let *v *be a node of *BFS*(*y*), then (*i*) holds by line 4. Now consider the case where *v *is strictly above *y *and *z*. We will prove (*ii*) by recursion on *v *following the order of the nodes in *BFS*(*y*). The recursion begins from the set of lowest nodes that are strictly above *y *and *z*, i.e. the set of nodes of *v *in $Min_{N}\left(y,\;z\right)$ such that there is not any node in $Min_{N}\left(y,\;z\right)$ that is below *v*. Let *v*_1_, *v*_2 _be two children of *v*, then by hypothesis *v*_1_, *v*_2 _are not strictly above *y*, *z*, i.e. *label*(*v*_1_) = *label*(*v*_2_) ≠ ∅; and *low*(*v*) = *v*. Thus, due to line 12, *label*(*v*) = *v *= *low*(*v*). Now let *v *be a node strictly above *y*, *z *such that (ii) is correct for each node below *v *which is strictly above or equal to *y, z*. If *v *is a hybridization node, then it is evident that *low*(*v*) = *low*(*v*_1_) where *v*_1 _is the only child of *v*. Moreover, since *label*(*v*_1_) = *low*(*v*_1_) (by the hypothesis of recurrence), then *label*(*v*) = *label*(*v*_1_) = *low*(*v*_1_) = *low*(*v*). If *v *is a speciation node having two children *v*_1_, *v*_2 _such that *v*_2 _is not in either *BFS*(*y*) or *BFS*(*z*), then we also have *low*(*v*) = *low*(*v*_1_). Hence, due to lines 5 - 6, we have *label*(*v*) = *label*(*v*_1_) = *low*(*v*_1_) = *low*(*v*). Now consider the last case, i.e. *v *is a speciation node having two children *v*_1_, *v*_2 _that are both in *BFS*(*y*) and *BFS*(*z*). If there exists a node *q *= *low*(*v*_1_) = *low*(*v*_2_), then *low*(*v*) = *q *because every from *v *to *y, z *must pass either through *v*_1 _or *v*_2_, i.e. always pass through *q*. Following line 10, we fix *label*(*v*) = *label*(*v*_1_) = *q *= *low*(*v*). Moreover, in this case *v *can not be in $Min_{N}\left(y,\;z\right)$ because every path from *v *to *y, z *passes through a node *q *below *v*. In the last case, we have $label\left(v_{\text{1}}\right)\neq label\left(v_{\text{2}}\right)$. Since both *v*_1_, *v*_2 _are above *y, z*, then there exists a node *q*_1 _= *label*(*v*_1_), and a different node *q*_2 _= *label*(*v*_2_). We will prove that $v\in Min_{N}\left(y,\;z\right)$. Indeed, suppose otherwise, then there is a node *q *such that every path from *v *to *y, z *must pass through *q*. Hence, every path from *v*_1 _(resp. *v*_2_) to *y, z *must also pass through *q*, so *q*_1 _*≤_N _q *(resp. *q*_2 _*≤_N _q*). It means that there is a path from *v*_1 _to *q *to *q*_2 _and then to *y, z *that does not pass through *q*_1_, a contradiction. Hence *v *is in $Min_{N}\left(y,\;z\right)$, and thus by definition the only node that every path from *v *to *y*, *z *must pass through is *v *itself (line 12).

We now present some intermediate results that will be useful to prove the complexity of Algorithm 2.

We extend the definition of $M_{N}$ to a subset of leaves. Let *L *be a subset of *L*(*N *). If |*L| *= 1, then $M_{N}\left(L\right)=L$. Otherwise, $M_{N}\left(L\right)$ is the set of nodes *m *of *N *such that *m *is above all leaves in *L *and there exist at least two separated paths from *m *to two distinct leaves of *L*.

Given a node *u *of *G*, *L_N _*(*u*) is defined as the set of leaves of *N *to which *α *maps the leaf set of *G_u_*, i.e. $L_{N}\left(u\right)=\{x\in L\left(N\right)|\exists u\in L\left(G_{u}\right)$ and *s*(*u*) = *s*(*x*)}.

**Lemma 6 ***Let α be a most parsimonious reconciliation between G and N, then, for every node u of *$G,\alpha_{r}\left(u\right)\in M_{N}\left(L_{N}\left(u\right)\right)$.

*Proof: *It is true for every leaf *u *of *G*. Let *u *now be an internal node having two children *u*_1_, *u*_2_.

If *u*_1_, *u*_2 _∈ *L*(*G*), then following Remark 1, *L_N _*(*u*) consists of two distinct nodes *α_r_*(*u*_1_), *α_r_*(*u*_2_). By Lemma 5, $\alpha_{e}\left(u\right)=S$ and $\alpha_{r}\left(u\right)\in Min_{N}\left(\alpha_{r}\left(u_{\text{1}}\right),\alpha_{r}\left(u_{\text{2}}\right)\right)$, i.e. there exist two separated paths from *α_r_*(*u*) to *α_r_*(*u*_1_) and *α_r_*(*u*_2_). It means that $\alpha_{r}\left(u\right)\in M_{N}\left(L_{N}\left(u\right)\right)$.

Let *u*_1 _∉ *L*(*G*), then following Remark 1, |*L_N _*(*u*_1_)| ≥ 2, then there always exist two distinct leaves *x, y *of *L_N _*(*u*) such that *x *∈ *L_N_*(*u*_1_), *y *∈ *L_N_*(*u*_2_), i.e. *x *is below *α_r_*(*u*_1_) and *y *is below *α_r_*(*u*_2_). If $\alpha_{e}\left(u\right)=S$, then following Lemma 5, $\alpha_{r}\left(u\right)\in Min_{N}\left(\alpha_{r}\left(u_{\text{1}}\right),\alpha_{r}\left(u_{\text{2}}\right)\right)$, i.e. there exist two separated paths from *α_r_*(*u*) to *α_r_*(*u*_1_), *α_r_*(*u*_2_). By extending these two paths from *α_r_*(*u*_1_) to *x*, and from *α_r_*(*u*_2_) to *y*, we have to two separated paths from *α_r_*(*u*) to *x, y*. In other words, *α_r_*(*u*) ∈ *ℳ_N _*(*L_N _*(*u*)). If *α_e_*(*u*) =  D and suppose that *α_r_*(*u*_2_) *≤_N _α_r _*(*u*_1_), then *α_r_*(*u*) = *α_r_*(*u*_1_) following Lemma 5, and *α_r_*(*u*) is not a leaf of *N *following Remark 1. Let *u' *be the highest node such that *u' *≤*_G _u*_1 _and $\alpha_{e}\left(u^{\prime }\right)=S$. Then following Lemma 5, $\alpha_{r}\left(u^{\prime }\right)=\alpha_{r}\left(u\right)$, and there exists two separated paths from *α_r_*(*u'*), i.e. from *α_r_*(*u*), to two distinct leaves of *L_N_*(*u'*), i.e. two distinct leaves of *L_N _*(*u*). We can prove the claim similarly for the case when *α_r_*(*u*_1_) *≤_N _α_r _*(*u*_2_).

**Lemma 7 ***If N is a network that contains h hybridization nodes, then for every subset L of $L\left(N\right),|M_{N}\left(L\right)|\leq h+\text{1}$ holds*.

The proof of this lemma is deferred to the appendix. We are now ready to state the complexity of Algorithm 2.

**Theorem 4 ***The time complexity of Algorithm 2 is O*(*h*^2^·|*G*|·|*N*|) *where h is the number of hybridization nodes of N*.

*Proof: *For every *u *∈ *V*(*G*), |C(u)| is equal to the possible nodes of *N *that *u *can be mapped to, which is bounded by $\left|M_{N}\left(L_{N}\left(u\right)\right)\right|$ by Lemma 6, and so by *O*(*h*) following Lemma 7.

The *for *loop at lines 3 - 23 is performed |*G| *times, and, at each iteration, the *for *loop at lines 10-23 is performed *O*(*h*^2^) times. In each iteration of the second loop, the operation *computeMin*, as detailed in Algorithm 3, requires two breath-first-search traversals, which can be performed in time *O*(|*N*|). Moreover, for every node *x *of $Min_{N}\left(y,\;z\right)$, by definition there exists two separated paths from *x *to *y, z*, which can be extended to be two separated paths from *x *to two distinct leaves *l*_1_, *l*_2 _of *L_N _*(*u*) where *l*_1 _is a leaf below *y *of and *l*_2 _is a leaf below *z*. This is always possible because, by Remark 1, |*L_N _*(*u*)| > 1. Hence, *x *must be in $M_{N}\left(L_{N}\left(u\right)\right)$ by definition, i.e. $Min_{N}\left(y,\;z\right)\subseteq M_{N}\left(L_{N}\left(u\right)\right)$, and thus $|Min_{N}\left(y,\;z\right)|\leq |M_{N}\left(L_{N}\left(u\right)\right)|\leq \left(h+\text{ 1}\right)$. Therefore, the loop at lines 13 - 16 can be performed in time *O*(*h*). The operation *merge*(*L*_1_, *L*_2_) at lines 23 for two lists of size *O*(*h*) can be implemented in times *O*(*h*), if we know that the resulting list is also of size *O*(*h*). Hence, it takes *O*(|*N*| + *h*) = *O*(|*N*|) times for each iteration of the loop 10 - 23. Therefore, the total complexity is *O*(*h*^2^· |*G*|·|*N*|).

Finally, a reconciliation of minimum cost between *G *and *N *can be then obtained by a standard back-tracking of the matrix  C, starting from any pair (*x,c*) of  C(*r*(*G*)) such that *c *is the minimum value over all pairs in  C(*r*(*G*)).

## Conclusions

In this paper, we have studied two variants of the reconciliation problem between a gene tree and a species network. In particular, for the problem of finding the "most parsimonious" switching of the network, even though the number of switchings can be exponential with respect to the number of hybridization nodes, we proposed an algorithm that is exponential only with respect to the level of the network, which is often low for biological data. Moreover, the problem of finding a reconciliation between a gene tree and a network, which was solved in [4] for a more general model but with a very high complexity, was re-studied here for a simpler model, which is more pertinent for same parts of the Tree of Life, and an algorithm with a much smaller complexity was provided. In a further work, we intend to implement the algorithms presented in this paper and apply them to biological data.

## Appendix

### Proof of lemma 3

By Definition 8, for every *u *∈ *I*(*G*), there must exist *H *∈ *G*_*B*(*u*) _such that *u *∈ *I*(*H*). If *H *is an edge, then *u *is the only internal node of *H*, which must be an artificial node by Lemma 2. But this is not possible because nodes of *G *cannot be artificial. Hence, *H *must be a binary tree. Let denote *B_i _*= *B*(*u*), and *S_i _*= *S*(*B*(*u*)). We will prove now that $\alpha \left(u\right)\;=\beta_{S_{i}}^{H}\left(u\right)$ by recursion on the height of *u*.

Let *u *be an internal node of *G *that has two children *u*_1_, *u*_2 _in *G*, and let *H *be the binary tree of $G_{B_{i}}$ such that *u *∈ *I*(*H*). Denote ${B_{i}}_{{}_{\text{1}}}=B\left(u_{\text{1}}\right)$, ${B_{i}}_{{}_{\text{2}}}=B\left(u_{\text{2}}\right)$, and ${S_{i}}_{{}_{\text{1}}}=S\left(B\left(u_{\text{1}}\right)\right)$, ${S_{i}}_{{}_{\text{2}}}=S\left(B\left(u_{\text{2}}\right)\right)$. For *j *= 1, 2, if *u_j _*is a leaf, let *H_j _*be equal to *u_j _*, otherwise *H_j _*is the binary tree of $G_{B_{i_{j}}}$ such that *u_j _*∈ *I*(*H_j_*). For the sake of convenience, if *u_j _*is a leaf, we also denote by $\beta_{S_{i_{j}}}^{H_{j}}$ the reconciliation that maps the only leaf *u_j _*to the only leaf *x *of *N *such that *s*(*x*) = *s*(*u_j _*). Note that *s*(*x*) = *α*(*u_j _*).

We now suppose that $\alpha \left(u_{j}\right)\;=\beta_{S_{i_{j}}}^{H_{j}}\left(u_{j}\right)$ for *j *= 1, 2 (which is evidently true if *u_j _*is a leaf), and we will show that this implies that the claim is true for *u*.

Let $u_{1}^{\prime }$(resp. $u_{2}^{\prime }$) be the child of *u *in *G_N _*such that $u_{\text{1}}\leq_{G_{N}}u_{1}^{\prime }{<}_{G_{N}}u\;\left(\text{resp}.{\;u}_{\text{2}}\leq G_{N}u_{2}^{\prime }\right)<G_{N}\;u)$. We respectively denote $B_{{i^{\prime }}_{1}}=B\left(u_{1}^{\prime }\right)$ and $B_{{i^{\prime }}_{2}}=B\left(u_{2}^{\prime }\right)$. By definition of the LCA reconciliation, we have $\beta_{S_{i}}^{H}(u)=LCA_{S_{i}}(\beta_{S_{i}}^{H}({u^{\prime }}_{1}),\beta ({u^{\prime }}_{2}))$.

(i) If ${B_{i}}_{{}_{\text{1}}}={B_{i}}_{{}_{\text{2}}}$, then $B_{i}=B_{i_{1}}=B_{{i^{\prime }}_{1}}=B_{i2}=B_{{i^{\prime }}_{2}}$, and *H *= *H*_1 _= *H*_2_. This implies that $u_{1}^{\prime }=u_{\text{1}}$, because otherwise *H *will contain an artificial node. The same holds for $u_{2}^{\prime }$ and *u*_2_. Thus, $\begin{array}{c} \alpha \left(u\right)=LCA_{S}\left(\alpha \left(u_{\text{1}}\right),\alpha \left(u_{\text{2}}\right)\right)=LCA_{S}\left(\beta_{S_{i_{1}}}^{H_{1}}\left(u_{\text{1}}\right),\beta_{S_{i_{2}}}^{H_{2}}\left(u_{\text{2}}\right)\right)\\ =LCA_{S}\left(\beta_{S_{i}}^{H}\left(u_{\text{1}}\right),\beta_{S_{i}}^{H}\left(u_{\text{2}}\right)\right)=LCA_{S_{i}}\left(\beta_{S_{i}}^{H}\left(u_{\text{1}}\right),\beta_{S_{i}}^{H}\left(u_{\text{2}}\right)\right)=LCA_{S_{i}}\left(\beta_{S_{i}}^{H}\left({u^{\prime }}_{\text{1}}\right),\beta_{S_{i}}^{H}\left({u^{\prime }}_{\text{2}}\right)\right)=\beta_{S_{i}}^{H}\left(u\right). \end{array}$

(ii) If ${B_{i}}_{{}_{\text{1}}}{<}_{N}{B_{i}}_{{}_{\text{2}}}$, then $B_{i}={B_{i}}_{{}_{\text{2}}}$, and *H *= *H*_2_. As in point (*i*), this implies $u_{2}^{\prime }=u_{\text{2}}$ and thus $\alpha \left(u\right)=LCA_{S}\left(\alpha \left(u_{\text{1}}\right),\alpha \left(u_{\text{2}}\right)\right)=LCA_{S}\left(\beta_{S_{i_{1}}}^{H_{1}}\left(u_{\text{1}}\right),\beta_{S_{i}}^{H}\left(u_{2}^{\prime }\right)\right)$. If $u_{1}^{\prime }=u_{1}$, then we have $LCA_{S}(\beta_{S_{i_{1}}}^{H_{1}}\left(u_{1}\right),\beta_{S_{i}}^{H}\left(u_{2}^{\prime }\right)=LCA_{S}(\beta_{S_{i}}^{H}\left(u_{1}^{\prime }\right),\beta_{S_{i}}^{H}\left(u_{2}^{\prime }\right)=\beta_{S_{i}}^{H}\left(u\right)$. Otherwise, $u_{1}^{\prime }$ is an artificial node which is a leaf of *H *that is mapped to *r*$\left(B_{i_{1}}^{\prime }\right)$ by $\beta_{S_{i}}^{H}$. By Lemma 1, $B_{i_{1}}\leq_{N}B_{{i^{\prime }}_{1}}{<}_{N}B_{i}$, so all nodes of *B_i _*are above all nodes of ${B_{i}}_{{}_{\text{1}}}$ and above $r\left(B_{{i^{\prime }}_{1}}\right)$. This implies that $LCA_{S}\left(\beta_{S_{i_{1}}}^{H_{1}}\left(u_{\text{1}}\right),\beta_{S_{i}}^{H}\left(u_{2}^{\prime }\right)\right)=LCA_{S}\left(r\left(B_{{i^{\prime }}_{1}}\right),\beta_{S_{i}}^{H}\left(u_{2}^{\prime }\right)\right)=LCA_{S_{i}}\left(\beta_{S_{i}}^{H}\left(u_{1}^{\prime }\right),\beta_{S_{i}}^{H}\left(u_{2}^{\prime }\right)\right)=\beta_{S_{i}}^{H}\left(u\right)$. Similarly for the case where ${B_{i}}_{{}_{\text{2}}}{<}_{N}{B_{i}}_{{}_{\text{1}}}$.

(iii) Suppose now that ${B_{i}}_{{}_{\text{1}}}$, ${B_{i}}_{{}_{\text{2}}}$ are not comparable and that $u_{1}^{\prime }\neq u_{\text{1}}$ and $u_{2}^{\prime }\neq u_{\text{2}}$ (the other cases can be shown reusing the arguments of point (*ii*)). Then, similarly as in point (*ii*), $B_{i_{1}}\leq_{N}B_{{i^{\prime }}_{1}}{<}_{N}B_{i},{B_{i}}_{{}_{\text{2}}}\leq_{N}B_{{i^{\prime }}_{2}}{<}_{N}B_{i}$, and $u_{1}^{\prime }$, $u_{2}^{\prime }$ are leaves of *H *mapped respectively to $r\left(B_{{i^{\prime }}_{1}}\right)$ and $r\left(B_{{i^{\prime }}_{2}}\right)$ by $\beta_{S_{i}}^{H}$. Since $\beta_{S_{i_{1}}}^{H_{1}}\left(u_{\text{1}}\right)$ is a node of ${B_{i}}_{{}_{\text{1}}}$, $\beta_{S_{i_{2}}}^{H_{2}}\left(u_{\text{2}}\right)$ is a node of ${B_{i}}_{{}_{\text{2}}}$, then $LCA_{S}\left(\beta_{S_{i_{1}}}^{H_{1}}\left(u_{\text{1}}\right),\beta_{S_{i_{2}}}^{H_{2}}\left(u_{\text{2}}\right)\right)=LCA_{S}\left(r\left(B_{{i^{\prime }}_{1}}\right),\;r\left(B_{{i^{\prime }}_{2}}\right)\right)=LCA_{S_{i}}\left(\beta_{S_{i}}^{H}\left({u^{\prime }}_{\text{1}}\right),\beta_{S_{i}}^{H}\left({u^{\prime }}_{\text{2}}\right)\right)=\beta_{S_{i}}^{H}\left(u\right).$

Therefore, in all cases we always have $\alpha \left(u\right)=\beta_{S}^{H}\left(u\right)$, and thus the same is true for every *u *∈ *I*(*G*) by recursion.

### Proof of lemma 7

**Figure 4:**
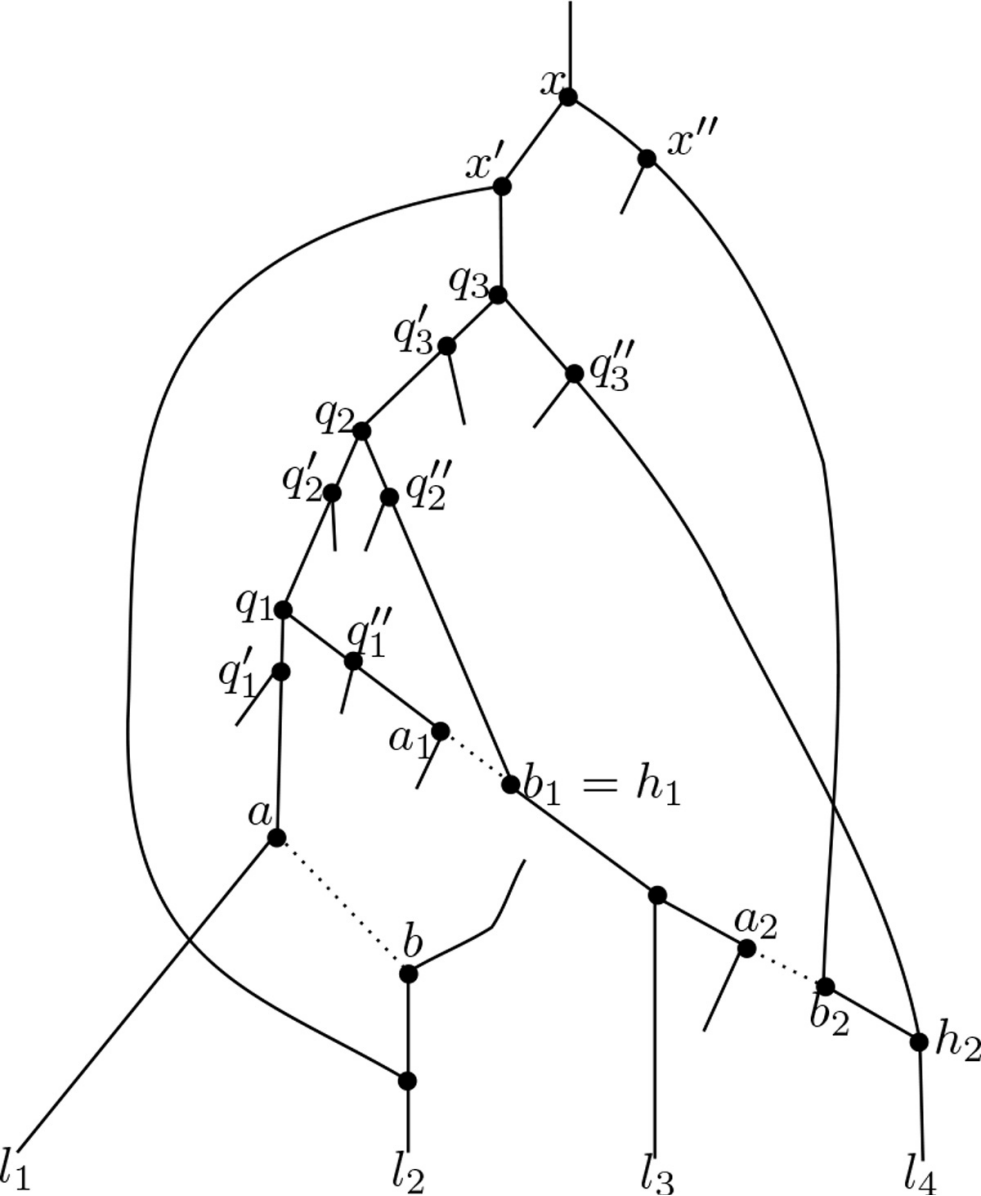
**An illustration for the proof of Lemma 7**. In this example: *L *= {*l*_1_, *l*_2_, *l*_3_, *l*_4_}, *Q *= {*q*_1_, *q*_2_, *q*_3_*}*, L^* ^= {*l*_1_, *l*_3_, *l*_4_}, *L*_3 _= {*l*_4_}, *L*_2 _= {*l*_3_}, and *L*_1 _= ∅. For *i *= 1, let *l*(*q*_1_) = *l*_3_; thus, we have *c*(1) = 2 and the path *p*_1 _starts from *q*_1_, passes through $q_{\text{1}}^{\prime \prime }$, *b*_1 _to *l*_3_, while the path $p_{1}^{\prime }$ starts from *q*_2_, passes through $q_{2}^{\prime \prime }$, *b*_1 _to *l*_3_. In this case, we have *h*_1 _= *b*_1_. For *i *= 2, let *l*(*q*_2_) = *l*_3_; thus we have *c*(2) = 3 and the path *p*_2 _starts from *q*_2_, passes through $q_{2}^{\prime \prime }$, *b*_1_, *b*_2_, *h*_2 _to *l*_4_, while the path $p_{2}^{\prime }$ starts from *q*_3 _passes through $q_{3}^{\prime \prime }$ to *l*_4_. Let *x *be a node in $M_{N^{\prime }}\left(L\right)$, then *x *is also in $M_{N^{\prime \prime }}\left(L\right)$ where *N" *is obtained from *N' *by removing all edges (*a_i_, b_i_*).

We will prove this lemma by recursion on the number of hybridization nodes. See the figure 4 for an example.

If there is no hybridization node, then *N *is a tree, and it is evident that $M_{N}\left(L\right)$ contains exactly one node.

Suppose that the claim is correct for every network having *h *hybridization nodes. Let *N *now be a network that has *h *+ 1 hybridization nodes. Let (*a, b*) be an edge of *N *having as target a hybridization node (namely, *b*) such that it does not exist any hybridization node above *a *(such a node always exists because *N *is a directed acyclic graph). Let *N' *be the network obtained by removing (*a, b*) from *N *(and also removing all nodes of indegree 1 and outdegree 1 created by this removal), then *N' *has exactly *h *hybridization nodes. Let $Q=M_{N}\left(L\right)\backslash M_{N^{\prime }}\left(L\right)$, then every node *q *in *Q *must be above *a*. Indeed, if *q *is not above *a*, then every path from *q *to every node of *L *does not contains (*a, b*), thus *q *is in $M_{N^{\prime }}\left(L\right)$, a contradiction. Moreover, by hypothesis, there is no hybridization node above *a*, hence all nodes of *Q *must be contained in a path leading *a*, and this path does not contain any hybridization to node. Let enumerate the nodes in *Q *as *q*_1_,... *q_m _*from the lowest to the highest one.

If $\left|Q\right|\leq \text{1},\text{then}|M_{N}\left(L\right)=|M_{N^{\prime }}\left(L\right)|+\left|Q\right|\leq h+\text{1}+\text{1}=h+\text{2}$, we are done.

Suppose now that |*Q| *= *m >*1. In the following, we will define *m - *1 edges of *N *having as target a hybridization node such that if *N" *is the network obtained from *N' *by removing these edges, then $M_{N^{\prime }}\left(L\right)=M_{N^{\prime \prime }}\left(L\right)$.

Denote by L^* ^the set of nodes in *L *that are below *q_m _*in *N'*. Hence L \ L^* ^is not empty since otherwise *q_m _*would be in $M_{N^{\prime }}\left(L\right)$, a contradiction. For every *q_i_*, *i *= 1,..., *m*, let $q_{i}^{\prime }$, $q_{i}^{\prime \prime }$ be the two children of *q_i _*such that $q_{i}^{\prime }$ is above or equal to *a*. Hence, $q_{i}^{\prime \prime }$, is not above or equal to *a *since there is no hybridization node above *a*, thus every path from *q_i _*to L \ L^* ^must pass through $q_{i}^{\prime }$ and *a, b*. By definition, there must exist at least two separated paths from *q_i _*to two leaves of *L*. Hence, for every *i*, there exists always a path from *q_i _*to a node of L^* ^that passes through $q_{i}^{\prime \prime }$. Denoted this node by *l*(*q_i_*).

Denote by *L_m _*the set of nodes in L^* ^such that, for every *l *∈ *L_m_*, there is a path from *q *to *l *that passes through $q_{m}^{\prime \prime }$. As explained above, *L_m _*is not empty. Recursively, for every *i< m*, let *L_i _*be the set of nodes in L^* ^\ *L*_*m *_\... *L*_*i*+1 _such that, for every *l *∈ *L_i_*, there is a path from *q*_*i *_to *l *that passes $q_{i}^{\prime \prime }$. Note that this set may be empty if *i< m*, and for every *i *≠ *j*, *L_i _*∩ *L_j_* = ∅.

We will define, for each *i < m*, an index *c*(*i*) that is strictly greater than *i *such that *L*_*c*(*i*) _≠ ∅, together with two paths *p_i _*(resp. $p_{i}^{\prime }$) from *q_i _*(resp. *q*_*c*(*i*)_) to a node of *L*_*c*(*i*) _as follows: if *l*(*q*_*i*_) is not in *L_i_*, then by the definition, there exists a unique *j *such that *L_j _*contains *l*(*q*_*i*_), and *j > i*. We fix *c*(*i*) = *j*. Next, we define *p_i _*(resp. $p_{i}^{\prime }$) as a path from *q_i _*(resp. *q*_*c*(*i*)_) to *l*(*q_i_*) that passes $q_{i}^{\prime \prime }$ (resp. ${q_{c}^{\prime \prime }}_{\left(i\right)}$). If *l*(*q_i_*) is in *L_i_*, then let *c*(*i*) be the smallest number that is greater than *i *and *L*_*c*(*i*) _≠ ∅ (such an index always exists because *L_m _*≠ ∅). Let *l' *be a node of *L*_*c*(*i*)_. Since *q_i _*is in *M_N _*(*L*), then there must exist a path from *q_i _*to *l'*, and we define *p_i _*as this path. The path $p_{i}^{\prime }$ is defined as the one from *q*_*c*(*i*) _to *l' *that passes through $q_{c\left(i\right)}^{\prime \prime }$. Note that *p_i_*, $p_{i}^{\prime }$ must contain at least one common hybridization node since they start from different nodes and end at a same leaf of *L*. Denote by *h_i _*the highest common hybridization node of *p_i _*and $p_{i}^{\prime }$. Hence, all *h_i_*s are distinct since each *p_i _*starts at a different node *q_i_*, and each $p_{i}^{\prime }$ starts at a node *q*_*c*(*i*) _that is strictly greater than *q_i_*. We define (*a_i_, b_i_*) recursively in increasing order of *i *from 1 to *m - *1 as follows. If *i *= 1, then *b_i _*is the highest hybridizatition node on *p_i_*. If *i >*1, then *b_i _*is the highest hybridization node on *p_i _*and different from all *b_k _*for every *k < i*. There exists always such a node *b_i_*, for example *h_i_*. Therefore, all *b_i_*s are distinct. Denote by *a_i _*the parent of *b_i _*on the path *p_i_*.

Denote by *N" *the network obtained from *N' *by removing all edges (*a_i_, b_i_*). For every node *x *in $M_{N^{\prime }}\left(L\right)$, we will prove that *x *is also in $M_{N^{\prime \prime }}\left(L\right)$. Denote by *x', x" *the two children of *x*. By definition, for every *l *∈ *L*, there exists a path, denoted by *f'*(*l*), in *N' *from *x *to *l *such that at least one path among them passes through *x'* and one other passes through *x"*. To prove that *x *is in $M_{N^{\prime \prime }}\left(L\right)$, we will now construct another set of paths in *N" *(i.e. in *N' *and does not contain any (*a_i_, b_i_*)) from *x *to each leaf *l *of *L*, denoted by *f"*(*l*), such that at least one path among them passes through *x'*and one other passes through *x"*.

Consider first the case that *x *is above *q_m _*(as shown in the figure 4). Without loss of generality, suppose that *x'* is above or equal to *q_m _*while *x" *is not. Suppose that *f'*(*l*) contains *q_m_*, then *l *∈ L^* ^and *f'*(*l*) must pass through *x' *because there is no hybridization node above *q_m_*. Suppose that $l\;\in L_{\text{1}}\cup ..\;.\;L_{m}$. Let *k *be the index such that *l *∈ *L_k_*, then we can choose a path *f"*(*l*) in *N*' from *x *to *l *that does not contain any (*a_i_, b_i_*) as follows. This path starts from *x*, passes through *x'*, goes down to *q_k _*, then takes the path from *q_k _*to *l *that contains *q"*. Note that this path does not include any *p_i _*since, by construction, every path *p_i _*starts from *q_i _*and goes to a node in *L*_*c*(*i*) _that is different from *L_i_*, while this path passes through *q_k _*and goes to a node in *L_k _*. Moreover, this path and *p_i _*can not have any common hybridization node above *a_i _*because *b_i _*is the highest hybridization node on *p_i_*. Hence, it can not pass through (*a_i_, b_i_*) for any *i*. If *l *is not in $L_{\text{1}}\cup ...\;L_{m}$, it means that every path from *q_m _*to *l *must pass through $q_{1}^{\prime }$, then we take the path starting from *x*, going down to $q_{i}^{\prime }$, then continuing to *l*. It is evident that this path does not include any *p_i_*, and it cannot have with *p_i _*any common hybridization node above *a_i _*because *b_i _*is the highest hybridization node on *p_i_*. Hence it does not pass through any (*a_i_, b_i_*). If *f'*(*l*) does not contain *q_m_*, then we fix *f"*(*l*) = *f'*(*l*). It is easy to see that *f'*(*l*) does not contain any edge (*a_i_, b_i_*) because otherwise *f'*(*l*) and *p_i _*must have at least a common hybridization node above *a_i _*(since there is no hybridization node above *q_i_*). But this is not possible because *b_i _*is the highest hybridization node on *p_i_*. Remark that at least one of the paths *f'*(*l*) in this case must pass through *x" *since all paths in the first case must pass through *x'*. Hence at least two of the paths *f"*(*l*) are separated, thus *x *is in $M_{N^{\prime \prime }}\left(L\right)$ by definition.

Consider now the case that *x *is not above *q_m_*, then similarly as in the previous case where *f'*(*l*) does not contain *q_m_*, we deduce that *f'*(*l*) does not contain any (*a_i_, b_i_*) for every *l*. Hence, by choosing *f"*(*l*) = *f'*(*l*) for every *l*, we are done.

Therefore, we have $M_{N^{\prime }}\left(L\right)=M_{N^{\prime \prime }}\left(L\right)$.

The network *N" *contains *h - |Q| *+ 1 hybridization nodes, then following the hypothesis of recurrence, $|M_{N^{\prime \prime }}\left(L\right)\leq h\;-|Q|+\text{ 2}$. This implies that $|M_{N^{\prime }}\left(L\right)|=|M_{N^{\prime \prime }}\left(L\right)\left|\leq h-\right|Q|+\text{ 2}$, thus $|M_{N}\left(L\right)|=|M_{N^{\prime }}\left(L\right)|+\left|Q\right|\leq h-|Q|+\text{2}+|Q|=h+\text{ 2}$.

## Competing interests

The authors declare that they have no competing interests.

## Authors' contributions

Both authors contributed to design the models, algorithms and to write the paper.
